# Temperature Sensor Assisted Lifetime Enhancement of Satellite Embedded Systems via Multi-Core Task Mapping and DVFS

**DOI:** 10.3390/s19224902

**Published:** 2019-11-09

**Authors:** Beomsik Kim, Hoeseok Yang

**Affiliations:** Department of Electrical and Computer Engineering, Ajou University, 206 Worldcup-ro, Yeongtong-gu, Suwon-si 16499, Korea; asd326541@ajou.ac.kr

**Keywords:** low earth orbit satellites, reliability, temperature sensors, real-time embedded systems, multi-core processor, dynamic voltage and frequency scaling (DVFS)

## Abstract

Recently, thanks to the miniaturization and high performance of commercial-off-the-shelf (COTS) computer systems, small satellites get popular. However, due to the very expensive launching cost, it is critical to reduce the physical size and weight of the satellite systems such as cube satellites (CubeSats), making it infeasible to install high capacity batteries or solar panels. Thus, the low-power design is one of the most critical issues in the design of such systems. In addition, as satellites make a periodic revolution around the Earth in a vacuum, their operating temperature varies greatly. For instance, in a low earth orbit (LEO) CubeSats, the temperatures vary from 30 to −30 degrees Celsius, resulting in a big thermal cycle (TC) in the electronic parts that is known to be one of the most critical reliability threats. Moreover, such LEO CubeSats are not fully protected by active thermal control and thermal insulation due to the cost, volume, and weight problems. In this paper, we propose to utilize temperature sensors to maximize the lifetime reliability of the LEO satellite systems via multi-core mapping and dynamic voltage and frequency scaling (DVFS) under power constraint. As conventional reliability enhancement techniques primarily focus on reducing the temperature, it may cause enlarged TCs, making them even less reliable. On the contrary, we try to maintain the TC optimal in terms of reliability with respect to the given power constraint. Experimental evaluation shows that the proposed technique improves the expected lifetime of the satellite embedded systems by up to 8.03 times in the simulation of Nvidia’s Jetson TK1.

## 1. Introduction

The last decade has witnessed dramatic growth of space industry; From 2010 to 2015, the nano/microsatellite market has grown at an annual average of 39%, and it is expected that, from 2016 to 2022, it will further grow at an annual growth of 13% [1]. Especially, the demand for small satellites has increased significantly as the space industry has shifted from the government to the private market. In keeping with such an increasing need for small satellites or space missions, the CubeSat standard was initiated [2] for small satellites that weigh about a few kilograms (In the standard, 1 unit is a 10 cm cube (10 × 10 × 10 cm${}^{3}$) with a mass of no more than 1.33 kg. A satellite may consist of a single (1U) or multiple cubes (3U, 6U, 12U and 27U).). While the CubeSats were originally developed for educational or demonstration purposes, their usages have been extended to more general and advanced missions, including scientific applications, deep space exploration, and so forth [3].

While the satellite systems are exposed to extreme conditions in terms of radiation and temperature, they are expected to operate for a long time without maintenance. Moreover, the missions imposed on such small satellites are getting more serious over time. In other words, the performance or reliability requirements of the onboard computer of satellite systems continue to increase. Thus, it is typical to design the satellite system with radiation-hardened processors [4] which generally have poorer performance than normal ones. In order to meet the reliability and performance requirements at the same time, the reconfigurable computing approach with field-programmable gate arrays (FPGAs) has been proposed, where various fault-tolerance techniques can be incorporated [5,6,7].

As CubeSats are subject to many physical constraints, including volume and weight, it is difficult to deploy large batteries or solar panels. Typical CubeSats with body-mounted solar panels generate less than 10 W, and state-of-the-art deployable solar panels produce 20–30 W. Batteries that are used in CubeSats typically store only 14–30 W·h [8]. In SwissCube [9], for instance, the average power generated from solar panels per orbit is only 1.5 W. Such a limited energy budget can restrict onboard computing performance. How to satisfy the increasing demand for performance and reliability within the given power budget is a challenge.

**Figure 1:**
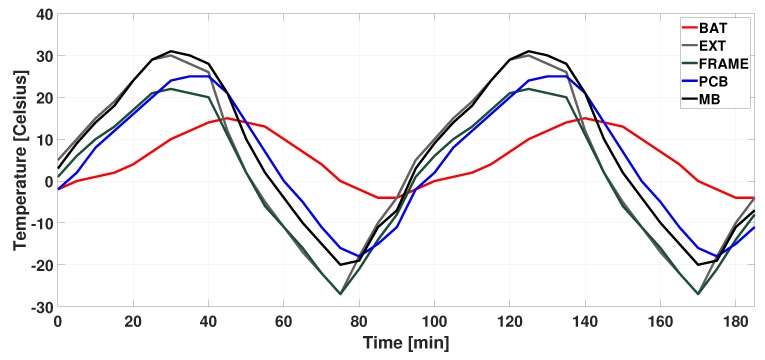
Temperature measurements in SwissCube [10] (BAT: battery, EXT: external, PCB: printed circuit board, and MB: motherboard).

Reliability is one of the key design concerns in a satellite. Most space missions require a long lifetime. In general, low earth orbit (LEO) satellites tend to have shorter expected lifetimes (5–10 years) than that of geostationary orbit (GEO) satellites (15 years or more). Since the maintenance is physically impossible in the satellite systems, it is essential to design them to operate for a long lifetime without any failure in the first place. The thermal cycling (TC) effect that satellite experience in extreme temperature changes is one of the major reliability threats. In a LEO CubeSat (SwissCube), for instance, external temperature is reported to change from 30${\;}^{\circ }C$ to −30${\;}^{\circ }C$ as illustrated in Figure 1 [10]. In order to keep the system intact in severe external temperature changes, many physical protections, including thermal control, multi-layer insulation, sun shields, radiators, heat pipes and so forth, are applied in high-end satellites. For the small satellites such as CubeSats, however, it is difficult to fully have such physical protections due to the cost and physical constraints.

In this paper, inspired by the fact that most small satellites are equipped with temperature sensors, we try to enhance the lifetime of the small satellite systems that are designed with multi-core processors without physical protections by adjusting the multi-core configuration in a temperature-aware manner. Thus far, most reliability enhancement techniques have tried to keep the operating temperature as low as possible [11,12,13] since it is well-known that high temperatures result in poor reliability. However, in satellite systems, this may not be the case as the external temperature varies greatly as shown in Figure 1. That is, in some cases, the artificial efforts to reduce the chip temperature may rather have an adverse effect of increasing the amplitude of TC.

To improve the lifetime reliability considering this TC effect, we propose to judiciously adjust the mapping of the software workload over the multiple cores and the operating frequency of the cores in a way that minimizes the amplitude of TC. In addition, we inject a virtual workload to the system if it is necessary to dissipate more power to improve the reliability. In doing so, the real-time schedulability of the satellites’ mission and power constraints should still be satisfied.

Our contributions can be summarized as follows:

- we identify the lifetime anomaly, where lower temperatures result in an even worse expected lifetime in satellite systems;
- then in order to mitigate the TC effects, identified above, we propose a mapping/frequency assignment technique for multi-core satellite systems.

In the proposed technique, we target the satellite systems implemented on top of homogeneous multi-core system, where each core can have an independent frequency/voltage configuration (While many commercially available multi-core platforms only support cluster-level frequency and voltage modulation, there are such flexible systems [14] and other reliability enhancement techniques including [15] also target the same architecture.). As workloads, we assume that the satellites software is implemented as a set of periodically invoked real-time tasks. In order to enable fast yet accurate temperature evaluations, we assume that task execution time is long enough to reach the steady-state temperature. We believe this assumption is reasonable thanks to the satellite systems’ low-power consumption. The inaccuracy that can be caused by this is analyzed in Section 4.4. To quantify the reliability of the system, we adopt the model proposed by Xiang et al. [16], where the lifetime is determined based on the temporal temperature profile. Considering the impact of spatial temperature gradients in the reliability remain as a future work.

The rest of this paper is organized as follows: In the next section, we discuss the related works and why the existing techniques are inefficient in the satellite systems. Section 3 quantitatively defines the proposed problem with task, architecture, power/temperature, and reliability models. Section 4 describes our method to maximize the lifetime reliability of the LEO satellite multi-core embedded systems in three steps. The evaluations are performed in simulation in Section 5 to show how the proposed technique improves the lifetime reliability, followed by concluding remarks and future works in Section 6.

## 2. Related Work

In addition to the TC effect that we mainly consider in this paper, there are three other known causes of failures in CMOS integrated circuits (ICs): electromigration (EM), time-dependent dielectric breakdown (TDDB), and stress migration (SM) [17]. Each of these failure mechanisms is quantified by Mean Time To Failure (MTTF), which is the expected lifetime concerning the failure source [18]. Most existing works focused on each of the above-mentioned causes individually, for example, EM [18,19,20], TDDB [18,21], SM [18], and TC [18,22]. Since these causes physically coexist in the operation of ICs, it is important to consider them altogether at the same time. Srinvasan et al. [23] proposed the Reliability-Aware Microprocessor (RAMP) model with negative bias temperature instability (NBTI) in addition to the four causes mentioned above. The five different failure causes are quantified in terms of reliability using the sum-of-failure-rates (SOFR) model, in which each failure mechanism is assumed to be associated with a constant failure rate. Xiang et al. [16] proposed a system-level reliability model with EM, TDDB, SM, and TC based on the Monte Carlo simulations.

It is well-known that high temperatures result in degradations in the IC’s lifetime [18,24,25]. So, based on a simple assumption that cooler ICs would always result in better reliability, many reliability enhancement techniques have been proposed to reduce the peak temperature without actually quantifying the expected reliability [11,12,13]. In these works, the actual reliability has not been quantitatively analyzed, but indirectly enhanced by reducing the temperature.

There are a handful of works that particularly focus on the TC effect. Ukhov et al. [26] proposed a multi-processor scheduling technique that maximizes the reliability considering the TC effect. While they showed that the MTTF could be improved by considering the TC effect in mapping/scheduling, the other causes, that is, EM, TDDB, and SM, were ignored in the reliability quantification. Rosing et al. [27] proposed the modified SOFR model and showed that aggressive power managements may harm the system’s reliability due to the TC effect. That is, dynamic power management (DPM) or dynamic voltage scaling (DVS) often causes temperature variations, and in some circumstances, these TC effects play crucial roles as the bottleneck in long-term reliability. Ma et al. [15] proposed an online framework that adjusts core frequencies and voltages in order to lower the peak temperature and balance the temperature differences between the cores in favor of the reduced TC effects. Chantem et al. [28] proposed a reliability-aware online task mapping/scheduling algorithm for homogeneous multi-core systems. They relied on a theoretical assumption that spatial and temporal load balancing would always improve the MTTF. However, to the best of our knowledge, none of the existing works takes the variable ambient temperatures into consideration except for Park et al. [29]. They proposed the dynamic thermal management (DTM) for networked embedded systems that consist of multiple vehicle electronic control units (ECUs) under high and variable ambient temperature. The proposed technique is different from their work in that it is focused on the TC effect caused by the repeating and highly varying ambient temperature of satellite systems. Further, the proposed technique tries to maximize the MTTF value directly, while Park et al. [29] indirectly enhance the reliability by reducing the peak temperature.

In this paper, we aim at maximizing the expected lifetime (MTTF) of satellite embedded systems that operate in the space environment where the temperature dynamically changes by an excessive amount as shown in Figure 1. We take this as a key technical challenge and propose a multi-core task mapping and dynamic voltage and frequency scaling (DVFS) technique that matches with such variable ambient temperature conditions using temperature sensors.

## 3. System Model

In this section, we describe the task, architecture, power/temperature, and reliability models, followed by the problem definition.

### 3.1. Task-Architecture Model

We consider a homogeneous multi-core system that consists of *M* cores, that is, $PE=\{pe_{1},pe_{2},\cdots ,pe_{M}\}$, as the target architecture. Each core can be operated at one of *L* different frequency levels, that is, $F=\{f_{1},f_{2},\cdots ,f_{L}\}$ and this frequency level can be modulated at runtime. Note that we assume that *F* is sorted in ascending order of frequency, that is, $\forall i<j,f_{i}<f_{j}$. The frequency selection of a core is defined as a function of fa:PE→F. For instance, when $pe_{m}$ is decided to be operated at $f_{l}$, $fa\left(pe_{m}\right)=f_{l}$. Also, it is assumed that a temperature sensor is placed on every core, thus, one can keep track of the temperature profile of each core.

For workloads on the target multi-core system, we consider an independent multi-task set that is defined as $W=\{\tau_{1},\tau_{2},\cdots ,\tau_{N}\}$. Each task is periodically invoked with a deadline. That is, $\tau_{n}$ is specified with a tuple $\left(ex_{n},p_{n}\right)$, where $ex_{n}$ and $p_{n}$ denote the number of worst-case execution cycles and the invocation period, respectively. Tasks have implicit deadlines, that is, the relative deadline of each invocation of $\tau_{n}$ is equal to $p_{n}$. In addition to the given workload *W*, we propose to inject a set of virtual tasks *V* if it is necessary to intentionally dissipate more heat in the system. Similarly, a virtual task $v_{i}\in V$ is also characterized by a tuple of the number of worst-case execution cycles and invocation period, that is, $\left(v\_ex_{i},v\_p_{i}\right)$.

We adopt the partitioned scheduling policy where the task-to-core assignment is defined as a function of map:(W∪V)→(PE∪{0}), that is, $map\left(\tau_{n}\right)=pe_{m}$ implies that task $\tau_{n}$ is executed on $pe_{m}$. If $map(\tau_{n})=0$, task $\tau_{n}$ is not mapped on any core. In the proposed technique, the mapping decision is made in two separate steps: task-to-logical-core mapping and logical-to-physical-core mapping. In the task-to-logical-core mapping represented by a function $map_{l}:\left(W\cup V\right)\to \left(LP\cup \left\{0\right\}\right)$, the workloads are mapped on $LP=\{lp_{1},\cdots ,lp_{M}\}$, a proxy of the physical cores PE. Then, the logical-to-physical-core mapping is determined by a function $map_{p}:LP\to \left(PE\cup \left\{0\right\}\right)$. For instance, if we have $map_{l}\left(\tau_{i}\right)=lp_{j}$ and $map_{p}\left(lp_{j}\right)=pe_{k}$, $map\left(\tau_{i}\right)=pe_{k}$. The assigned frequency of a logical core is preserved in the physical core, that is, $fa\left(lp_{i}\right)=fa\left(pe_{j}\right)$ if $map_{p}\left(lp_{i}\right)=pe_{j}$. Note that the execution time of a task is dependent upon the frequency assignment decision. If $\tau_{n}$ is assigned to core $pe_{m}$, the worst-case execution time of one invocation of $\tau_{n}$ is $ex_{n}/fa\left(map\left(\tau_{n}\right)\right)$. Then, the initial utilization of core $pe_{m}$ at the lowest frequency can be calculated as $u_{m}=\sum_{\tau_{i}\;s.t.\;map\left(\tau_{i}\right)=pe_{m}}\frac{ex_{i}}{p_{i}\cdot f_{1}}$. With the frequency modulation, $f_{1}$ in the equation can be replaced with $fa(pe_{m})$. Once the mapping decision is made, the multiple tasks on each core are scheduled according to the preemptive earliest-deadline-first (EDF) policy.

### 3.2. Power-Temperature Model

The power consumption at time *t* can be characterized as follows:

(1) $$P\left(t\right)=U\circ P_{act}\left(f\right)+P_{oth}\left(f\right)+P_{leak}\left(T\left(t\right)\right).$$

Note that we keep track of the temperature of *M* cores, thus, P(t) is the M×1 vector and so are $P_{act}\left(f\right)$, $P_{oth}\left(f\right)$, and $P_{leak}\left(T\left(t\right)\right)$. f is the M×1 frequency vector that indicates the current frequency assignment of each core, that is, $f={\left[fa\left(pe_{1}\right),fa\left(pe_{2}\right),\cdots ,fa\left(pe_{M}\right)\right]}^{\prime }$. Likewise, U is the utilization vector of *M* cores, that is, $U={\left[u_{1},u_{2},\cdots ,u_{M}\right]}^{\prime }$. $P_{act}\left(f\right)$ is the active power consumption vector when all cores are utilized by 100% at the frequency assignment of f, so, the element-wise multiplication $U\circ P_{act}\left(f\right)$ accounts for the active power consumption of the system under the current load. $P_{oth}\left(f\right)$ is the utilization-independent dynamic power consumption vectors, while $P_{leak}\left(T\left(t\right)\right)$ is the temperature-dependent leakage power consumption vectors.

While the CMOS power consumption is usually modeled as a simple summation of active and static power consumption, we elaborate on the modeling of temperature-dependent static power, using $P_{leak}$, as we target the satellite system where temperature-dependent leakage could be crucial. In fact, leakage power becomes increasingly significant in CMOS IC due to the technology scaling and it has been reported that it accounts for up to 40% of the power consumption of today’s microprocessors [30]. We use the piece-wise linear (PWL) leakage model which is known to be fast and highly-accurate [30,31] as follows:

(2) $$P_{leak}\left(T\left(t\right)\right)=\alpha \cdot T\left(t\right)+\beta .$$

where α and β are *M* × *M* and *M* × 1 fitting coefficient diagonal matrix/vector, respectively.

For temperature evaluations, we rely on the thermal RC-circuit model for multi-core systems [32], which is based on the duality between heat transfer and electrical phenomena. In that RC-circuit model, electrical current and capacitance corresponds to heat flow through the (thermal) resistance and the heat-absorbing capability of the component, respectively [33]. In that model, the temperature of a certain position can be easily obtained by evaluating the voltage in the circuit. To be more specific, we use the following equation for evaluating the M×1 temperature vector T(t):

(3) $$C\cdot \frac{dT(t)}{dt}=P\left(t\right)+K\cdot T_{amb}-\left(G+K\right)\cdot T\left(t\right).$$

In the above equation, C denotes the thermal capacitance of the *M* cores, represented as M×M diagonal matrix. Heat transfer between cores is taken into account by G, while heat dissipation from cores to the outside is by K. Note that both G and K are M×M thermal conductance matrices, and K is a diagonal matrix. $T_{amb}$ is the M×1 temperature vector that denotes the ambient (environment) temperature and P(t) is the power consumption vector formulated in Equation (1).

Equation (3) can be simplified as follows, using $A=C^{-1}\cdot (G+K-\alpha$), $B=C^{-1}\cdot \left(\beta^{\prime }+K\cdot T_{amb}\right)$, and $\beta^{\prime }=U\circ P_{act}\left(f\right)+P_{oth}\left(f\right)+\beta$:

(4) $$\frac{dT(t)}{dt}=-A\cdot T\left(t\right)+B.$$

When the system keeps the same configuration, that is, mapping and frequency assignment, for a long enough time, it will eventually reach a steady-state. The steady-state temperature can be easily obtained by having $\frac{dT(t)}{dt}=0$ in Equation (4). That is, the steady-state temperature vector $T_{ss}$ can be formulated as follows

(5) $$T_{ss}=A^{-1}\cdot B={\left(G+K-\alpha \right)}^{-1}\cdot \left(\beta^{\prime }+K\cdot T_{amb}\right).$$

Solving the differential Equation (4), we have the temperature vector

(6) $$T\left(t\right)=T_{ss}+\left(T\left(t_{0}\right)-T_{ss}\right)\cdot e^{-A(t-t_{0})}.$$

where $T(t_{0})$ is initial temperature vector.

### 3.3. Reliability Model

Among the four failure mechanisms we consider in this work, EM, TDDB, and SM can be modeled as follows in Equations (7)–(9) [16]:

(7) $$MTTF_{EM}=\frac{A_{EM}}{J^{n}}\cdot e^{\frac{E_{a,EM}}{k\cdot T}},$$

(8) $$MTTF_{TDDB}=A_{TDDB}\cdot {\left(\frac{1}{V}\right)}^{\left(a-bT\right)}\cdot e^{\frac{X+Y/T+ZT}{k\cdot T}},$$

and

(9) $$MTTF_{SM}=A_{SM}\cdot {\left|T_{o}-T\right|}^{-n}\cdot e^{\frac{E_{a,SM}}{k\cdot T}}.$$

What those three models have in common is that they are strongly dependent on the *absolute degree* of the temperature. That is, simply, the higher *T* they have, the smaller the MTTF values are. On the other hand, TC exhibits a different behavior as the wear in TC is mainly due to the difference in thermal expansion coefficients between adjacent material. This accumulated damage causes permanent failure in the package, solder, interconnects, and dielectric materials. Thus, in TC, how much *temporal temperature gradient* a system has is important. The MTTF due to TC can be modeled as follows [16]:

(10) $$MTTF_{TC}=\frac{p}{\sum_{}^{}\frac{1}{N_{C_{i}}}}$$

with *p* equal to the period of temperature history. $N_{C_{i}}$ is the effect of cycle *i* and can be quantified by the modified Coffin-Manson equation with the Arrhenius term [16] as follows:

(11) $$N_{C_{i}}=A_{TC}\cdot {\left(\Delta T-\Delta T_{0}\right)}^{-q}\cdot e^{\frac{E_{a,TC}}{k\cdot T_{max}}}$$

where $A_{TC}$ is fitting constant, ΔT is cycle amplitude, $\Delta T_{0}$ is the portion of the temperature range in the elastic region, $E_{a,TC}$ is activation energy, *k* is Boltzmann’s constant, $T_{max}$ is the maximum temperature during the cycle, and *q* is Coffin-Manson exponent constant that depends on the material characteristic. Usually, *q* is set to 6–9 for brittle fracture (Si and dielectrics), to 3–5 for hard metal alloys/intermetallics (Al-Au), and to 1–3 for ductile metal (solder) [18]. It is worthwhile to mention that in the TC mechanism, unlike all others, the negative impact of temperature gradient ΔT is explicitly considered, which motivates our work.

### 3.4. Problem Definition

The problem we target to solve in this work can be summarized as follows:

**Input:** Given the LEO CubeSat PCB temperature history as exemplified in Figure 1, that is, ambient temperature ($T_{amb}$), the periodic task set *W* as workloads, and the power-temperature models presented in Section 3.2,

**Constraints:** while respecting the timing constraints of the given task sets ($p_{i}$ for each $\tau_{i}\in W$) and not violating the given power budget $P_{max}$, that is, $\forall t,\sum_{i=0}^{M}P\left(t\right)\left[i\right]\leq P_{max}$,

**Output:** determine the mapping decision map and the frequency assignment fa, and impose additional virtual workload *V* and determine its mapping if necessary,

**Objective:** in order to maximize the MTTF.

## 4. Proposed Mapping/DVFS Technique

In this section, we propose a mapping/DVFS technique for multi-core embedded systems, presented in Section 3, tailored to the reliability optimization of LEO satellites. The most noticeable property of the target system is that they are exposed to highly varying temperature environments as shown in Figure 1. Note that most conventional approaches typically try either to minimize the power consumption or to keep the temperature as low as possible. In the highly varying temperature condition, however, such approaches may result in considerable temperature fluctuations over time, which, in turn, can negatively affect the system as a reliability threat.

We argue that the temperature management decision should be judiciously made considering the outside temperature obtained through a temperature sensor. For instance, when the environment temperature is very low, it would be even better to have higher clock frequencies than necessary in order to intentionally heat up the cores. This over-clocking decision is helpful to reduce the amplitude of the TC. On the other hand, this is not always feasible or desirable. Firstly, the power budget may now allow wasteful over-clocking in some cases. Or, if the outside temperature is relatively high, this may cause even bigger thermal gradients. It is also important to consider the three other failure mechanisms as well as TC. Therefore, it is not trivial to make an optimal mapping/DVFS decision for the given condition.

**Figure 2:**
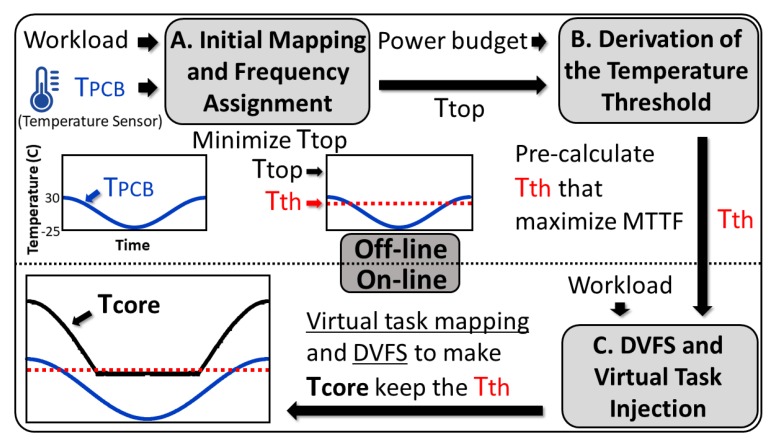
Overall procedure of the proposed mapping/DVFS technique.

Whilst the cycle of revolution of the LEO satellites is consistent, the maximum and minimum temperatures within the cycle vary depending on the season. Therefore, it is computationally intractable to precompute the mapping/DVFS solutions for all possible conditions. We propose a hybrid solution, that consists of two offline steps followed by an online step, as outlined in Figure 2. First, at the highest temperature of the revolution cycle, an initial mapping/DVFS decision is made in a way that minimizes the peak temperature ($T_{top}$). How to obtain this initial mapping is presented in Section 4.1. Then, out of this initial condition, the minimum temperature of the TC that causes the largest MTTF value is derived as a temperature threshold ($T_{th}$). This procedure is described in Section 4.2. At runtime, whenever this threshold is violated, that is, the temperature goes below the derived minimum, a new DVFS decision is made and a set of a virtual task is injected if necessary (Section 4.3).

### 4.1. Initial Mapping and Frequency Assignment

The main idea of the initial mapping and frequency assignment is to minimize the peak temperature as it is at the highest environment (PCB) temperature. In doing so, we rely on the worst-fit (WF) heuristic which has also been popularly used in the existing multi-core mapping approaches [28,34]. The initial mapping consists of three sub-procedures: (i) task-to-logical-core mapping; (ii) frequency modulation; and (iii) logical-to-physical-core mapping.

Algorithm 1 delineates the initial mapping procedure. Firstly, it determines the task-to-logical-core mapping in a way that the workloads are evenly distributed over the all constituent cores by means of the WF bin-packing heuristic (lines 1–6). After sorting out the tasks in descending order of utilization (line 1), it maps the tasks one by one to the idlest logical core (lines 4–5). Once the logical mapping is done, it scales up the operating frequencies of the cores as necessary (lines 8–16). That is, if a core is used by too many tasks exceeding its limit (line 9), its frequency is scaled up by one level until the utilization gets less than or equal to 1.0. With these frequency assignments done, we can calculate the power consumption of each core excluding the temperature-dependent part (line 17). At last, the logical-to-physical-core mapping decision is made in the third part (lines 19–34), in which the maximum steady-state temperature of the system is supposed to be minimized. In doing so, the logical core with the maximum power consumption that remains unmapped is chosen (line 21). Then, all possible physical core mapping candidates are investigated (lines 23–30) and the one that results in the lowest maximum steady-state temperature is chosen for mapping (line 31).

**Algorithm 1** Initial Mapping and Frequency Assignment
1:sort *W* in a descending order of $\frac{ex_{n}}{p_{n}\cdot f_{1}}$;2:∀n, set $map_{l}\left(\tau_{n}\right)=0$                    ▹ (i) Logical core mapping3:**for**n=1 to *N* **do**4:    find $lp_{m}$ with the minimum $u_{m}$ value;5:    set $map_{l}\left(\tau_{n}\right)=lp_{m}$ and update $u_{m}$;6:
**end for**
7: 8:**for**m=1 to *M* **do**                     ▹ (ii) Frequency modulation9:    **while** $u_{m}>1$ **do**10:        **if** $fa\left(lp_{m}\right)=f_{L}$**then**                     ▹ Highest frequency11:           return not schedulable;12:        **else**                     ▹ Scaling up the frequency by one level13:           when $fa\left(lp_{m}\right)=f_{l}$, adjust $fa(lp_{m})$ to $f_{l+1}$;14:        **end if**15:    **end while**16:
**end for**
17:calculate P with Equation (1) (w/o $P_{leak}$)18: 19:∀i, set $map_{p}\left(lp_{i}\right)=0$;                  ▹ (iii) Physical core mapping20:
**while** LP≠ϕ **do**
21:    find $lp_{i}\in LP$ with the maximum P[i];22:    $T_{curr\_min}\leftarrow \infty$; ind_min←1;23:    **for** j=1 to *M* **do**                     ▹ Find the smallest temp24:        set $map_{p}\left(lp_{i}\right)=pe_{j}$;                  ▹ Try mapping $lp_{i}$ on $pe_{j}$25:        evaluate the maximum temperature $T_{max}$;26:        **if** $T_{max}<T_{curr\_min}$ **then**27:           $T_{curr\_min}\leftarrow T_{max}$; ind_min←j;28:        **end if**29:        set $map_{p}\left(lp_{i}\right)=0$;                    ▹ Restore the mapping30:    **end for**31:    set $map_{p}\left(lp_{i}\right)=pe_{ind\_min}$;32:    $LP\leftarrow LP-\{lp_{i}\}$;33:
**end while**
34:return schedulable;


### 4.2. Derivation of the Temperature Threshold

Once the initial mapping/DVFS decision is fixed, we derive the temperature threshold vector $T_{th}$, above which the target system is always kept at runtime. For that, we first derive a model temperature profile $T_{ev}$ whose minimum temperature is $T_{th}$. The main challenge in finding a good $T_{ev}$ is to maximize the MTTF value considering TC and other three failure sources within the given power budget, $P_{max}$.

**Figure 3:**
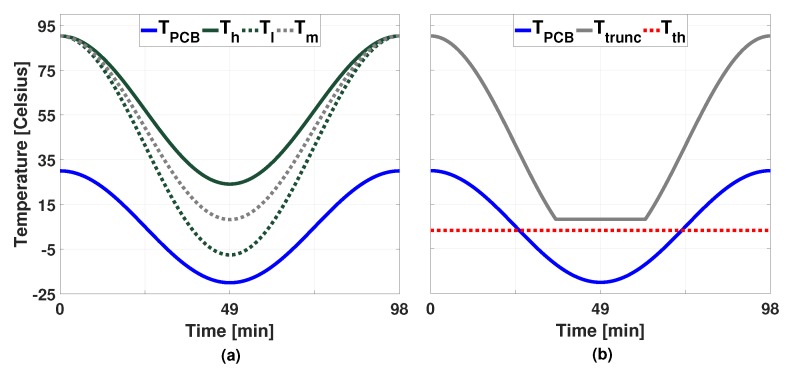
(**a**) Example temperature traces compared in the binary search and (**b**) the truncated envelope temperature and the temperature threshold.

The procedure of finding $T_{ev}$ is as follows. From the initial mapping, we can already determine the highest temperature vector, denoted as $T_{top}$, in a single revolution cycle. Basically, we iterate a number of candidates for the lowest temperature vector, $T_{bot}$, by means of binary search to find the one that results in the largest MTTF value. In order to limit the search range ($T_{bot,min}\leq T_{bot}\leq T_{bot,max}$), we lower-bound $T_{bot}$ by $T_{bot,min}$ which can be obtained by invoking Algorithm 1 at the lowest ambient temperature ($T_{PCB}$ in Figure 3). Similarly, we set the upper-bound of $T_{bot}$ as $T_{bot,max}$, which can be obtained by assuming that every core is utilized by 100% at the highest frequency with the same mapping. Once those maximum and minimum temperatures, $T_{top}$ and $T_{bot}$ are fixed and the intermediate temperatures between the two can be interpolated using the cosine function as follows (Note that the temperature changes caused by highly varying ambient temperature are modelled by a cosinusoidal form based on the observation of the temperature measurement data from SwissCube [10].):

(12) $$T_{ev}\left(t\right)=\frac{T_{top}-T_{bot}}{2}\cos\left(\frac{2\pi t}{t_{p}}\right)+\frac{T_{top}+T_{bot}}{2}$$

where $t_{p}$ denotes the revolution period of the satellite.

The first half of Algorithm 2 (lines 1–23) illustrates this binary search procedure. Note that we borrow the Monte-Carlo simulation framework from Xiang et al.’s work [16] for the evaluation of MTTF for a temperature profile T and this is denoted as SIM(T) in the pseudocode. It is worthwhile to mention that the temperature profile used during the binary search is not the exact one. It is an approximated one that is temporarily used for quantifying the effect of candidate TCs. Figure 3a demonstrates exemplary temperature profiles that are compared in the binary search.

Note in Equation (11) that TC is only dependent on the peak and bottom temperatures of the cycle, not on the intermediate temperatures in-between. Thus, regarding the TC effect, just keeping the system temperature above $T_{th}$ is fine. Considering the other three effects, it is desirable to minimize power consumption to reduce the temperature. So, we keep the mapping/DVFS decision obtained by Algorithm 1 as long as the temperature threshold is not violated. On the other hand, if this threshold is too high, it would not be feasible to heat up the system within the given power budget. So, in the second half of the algorithm (lines 25–30), $T_{th}$ is adjusted to confirm that the threshold is always maintainable with the given power budget. The expected power consumption is calculated using the modified Equation (5): $P\left(t\right)=\left(G+K\right)\cdot T_{}\left(t\right)-K\cdot T_{amb}$. That is, we calculate back the required power P(t) that results in the steady-state temperature of T(t). If it violates the following power constraint, $\forall t,\sum_{i=0}^{M}P_{ev}\left(t\right)\left[i\right]=P_{ev}^{sum}\leq P_{max}$, it repeatedly reduces the temperature threshold (line 29) until satisfied.

**Algorithm 2** Derivation of the Temperature Threshold
1:$T_{h\_bot}\leftarrow T_{bot,max}$                       ▹ Initialization2:$T_{l\_bot}\leftarrow T_{bot,min}$;3:$T_{th}\leftarrow T_{l\_bot}$;4:$T_{h}\left(t\right)\leftarrow$ Equation (12) using $T_{bot}=T_{h\_bot}$;5:$T_{l}\left(t\right)\leftarrow$ Equation (12) using $T_{bot}=T_{l\_bot}$;6:$MTTF_{h}\leftarrow$SIM($T_{h}$), $MTTF_{l}\leftarrow$SIM($T_{l}$);7: 8:**while** true **do**                         ▹ (i) Binary search9:    $T_{m\_bot}\leftarrow \left(T_{h\_bot}+T_{l\_bot}\right)/2$;10:    $T_{m}\left(t\right)\leftarrow$ Equation (12) using $T_{bot}=T_{m\_bot}$;11:    $MTTF_{m}\leftarrow$SIM($T_{m}$);12:    **if** $MTTF_{h}\geq MTTF_{l}$ **then**                ▹$T_{bot}$ to be increased13:        $T_{l\_bot}\leftarrow T_{m\_bot}$, $MTTF_{l}\leftarrow MTTF_{m}$;14:        **if** $T_{h\_bot}≃T_{m\_bot}$ **then**15:           $T_{th}\leftarrow T_{h\_bot}$; **break**;16:        **end if**17:    **else**                           ▹$T_{bot}$ to be decreased18:        $T_{h\_bot}\leftarrow T_{m\_bot}$, $MTTF_{h}\leftarrow MTTF_{m}$;19:        **if** $T_{l\_bot}≃T_{m\_bot}$ **then**20:           $T_{th}\leftarrow T_{l\_bot}$; **break**;21:        **end if**22:    **end if**23:
**end while**
24: 25:$T_{trunc}\left(t\right)\leftarrow$ Equation (12) using $T_{bot}=T_{bot,min}$;26:**repeat**                           ▹(ii) Power constraint27:    ∀t s.t. $T_{trunc}\left(t\right)<T_{th}$, $T_{trunc}\left(t\right)\leftarrow T_{th}$;28:    calculate $P_{ev}^{sum}$ with $T_{trunc}\left(t\right)$;29:    $T_{th}\leftarrow T_{th}-{\left[1,\cdots ,1\right]}^{\prime }$;30:**until**$P_{max}\geq P_{ev}^{sum}$;31:return $T_{th}$;


### 4.3. DVFS and Virtual Task Injection

At runtime, the initial decision made by Algorithm 1 is preserved unless the temperature threshold is violated. In case of the violation, the system needs to artificially heat up properly. On the occasion of the $T_{th}$ violation, Algorithm 3 is invoked. It first reads the current temperatures $T_{cur}$ from the temperature sensors (line 1) and calculates the current power vector $P_{cur}$ (line 2). At line 3, the power consumption needed to reach the threshold temperature $T_{th}$ is calculated. Then, for each core (line 5), it tries to scale up the frequency repeatedly (lines 7–8) until it either reaches to the maximum level (line 7) or the target power consumption is reached (line 6). When a core frequency is scaled up maximally and the target power consumption is not reached yet (line 9), a virtual task $v_{i}$ is injected on the core (lines 11–12). Note that the execution cycle and period of the virtual task is fixed a priori.

As invoked at runtime, it is important for Algorithm 3 to be light-weight to be executed without causing considerable overheads in CPU. The time complexity of Algorithm 3 is O(|M|·L), where *L* denotes the maximum number of iterations of the while loop in lines 6–19. We believe that both |M| and *L* are manageably small for the following reasons. First, the number of cores installed in the small satellite, |M|, is typically small. And, the maximum number of the while loop iterations, *L*, is also not too big due to the triggering condition of Algorithm 3. Note that Algorithm 3 is invoked *each* time the $T_{th}$ violation is detected. Thus, the current power that causes the temperature violation ($P_{th}\left[m\right]$) is not so far away from ($P_{cur}\left[m\right]$). Thus, the actual number of loop iterations is not usually big. From our empirical evaluations, that will be presented in Section 5, the maximum number loop iterations caused by the frequency scaling (lines 7–8) and virtual task injection (lines 9–17) were only 2 and 5, respectively.

**Algorithm 3** DVFS and Virtual Task Injection
1:$T_{cur}\leftarrow$ current temperature sensor values;2:Calculate $P_{cur}$ with $T_{cur}$ using Equation (1);3:$P_{th}=\left(G+K\right)\cdot T_{th}-K\cdot T_{amb}$;4:i←0;5:**for**m=1 to *M* **do**                        ▹ For each core6:    **while** $P_{th}\left[m\right]>P_{cur}\left[m\right]$ **do**7:        **if** $fa\left(pe_{m}\right)\neq f_{L}$**then**                   ▹ Frequency scaling8:           when $fa\left(pe_{m}\right)=f_{l}$, $fa(pe_{m})$ to $f_{l+1}$;9:        **else**                          ▹ Virtual task injection10:           **if** $u_{m}+\frac{v\_ex_{}}{v\_p_{}\cdot f_{L}}\leq 1$ **then**11:               $V\leftarrow V\cup \{v_{i}\}$;12:               set $map\left(v_{i}\right)=pe_{m}$ and update $u_{m}$;13:               i←i+1;14:           **else**15:               break;16:           **end if**17:        **end if**18:        update $P_{cur}\left[m\right]$;19:    **end while**20:
**end for**



### 4.4. Inaccuracy of Temperature Analysis

In Algorithm 2 and 3, the temperature is not exactly evaluated, but approximately assuming a steady-state. In some cases, task execution time is not long enough to reach the steady-state temperature. The exact transient temperature can only be calculated by Equation (6), which is computationally too expensive. Therefore, we adopt the steady-state temperature approximation (line 28 in Algorithm 2 and line 3 of Algorithm 3). To compensate for the inaccuracy caused by the approximation, we introduce a margin $P_{\Delta }$ in the temperature comparison. That is, the inequality at line 3 of Algorithm 3 can be replaced with $P_{th}\left[m\right]+P_{\Delta }>P_{cur}\left[m\right]$. It is also worthwhile to mention that there could be a lot of small TCs in an actual schedule as the task execution bursts and idle times are interleaved in a complicated pattern. However, we decide to ignore the effect of such small cycles by approximating the temperature evaluation as it has been reported that they do not cause any serious long-term reliability problems [27,35].

In summary, Algorithm 1 minimizes the peak temperature of the core at the highest PCB temperature to reduce TC amplitude. Algorithm 2 derives the minimum temperature (threshold), above which the system’s temperature should be maintained, considering the power budget. Algorithm 3 artificially heats up the processor by means of DVFS and virtual task injections, whenever the temperature goes below the threshold.

## 5. Experiments

### 5.1. Evaluation Environment and Parameters

For evaluation, we choose Nvidia’s Jetson TK1 as the target architecture, which has quad-core ARM Cortex-A15 CPU and supports 12 different frequencies from 1.24 to 2.32 GHz, that is, $PE=\{pe_{1},pe_{2},pe_{3},pe_{4}\}$ and F={1.24,1.33,1.43,1.53,1.63,1.73,1.84,1.94,2.01,2.12,2.22,2.32}. Though all cores operate at the same frequency in the actual setup of the target architecture, we assume that each core can have an independent frequency level in the simulation. (Ma et al. [15] also had the same assumption in their evaluations.)

We borrow the power and temperature parameters of Nvidia’s Jetson TK1 from Ma et al.’s work [15] to characterize the power and temperature behaviors. For the temperature-dependent leakage power, we extend their power model using the PWL approximation [30]. And, the model is calibrated to have the same power consumption reported in Reference [15] at the ambient temperature of 20 ${}^{\circ }C$ using the leakage power estimation, presented in Reference [31]. The resultant power model and its parameters are as follows: $P_{act}\left(f\right)=0.8031\cdot f^{2}-2.046\cdot f+1.481$, $P_{oth}\left(f\right)=-0.08089\cdot f^{2}+0.3841\cdot f$, and $P_{leak}\left(T\left(t\right)\right)=\alpha \cdot T\left(t\right)+\beta$, where (α, β) is (0.001796,0.1098) if T(t)<0, (0.00393,0.1079) if 0≤T(t)<40, (0.006781,−0.0080065) if 40≤T(t)<80, and (0.01035,−0.2955) if T(t)≥80. For all cores, thermal capacitance *C* and thermal ground conductance *K* is set to 2.34 $J{/}^{\circ }C$ and 0.098 $W{/}^{\circ }C$, respectively. The thermal conductance *G* to the adjacent cores and to the core are set to −0.03 $W{/}^{\circ }C$ and −0.0075 $W{/}^{\circ }C$, respectively, for all cores. To obtain the temperature profile with respect to the given power profile, the architecture-level thermal RC-circuit with the above parameters is evaluated. In doing so, Equation (6) is calculated with the given parameters for each time step in MATLAB.

In the reliability model, since we consider the failure of the brittle materials [18,36,37], we set the Coffin-Manson exponent *q* to 6 in the Monte Carlo simulator [16]. All other parameters of the four mechanisms were set to the default values.

We modeled a set of periodic tasks using actual satellite workload periodic profiles such as executive, attitude determination and control, thermal management, and power management software, that is, $W=\{\tau_{1},\tau_{2},\cdots ,\tau_{9}\}$, the tuple denoted as $\left(\frac{ex_{n}}{f_{1}},p_{n}\right)$, that is, $\tau_{1}=\left(0.06,0.1\right)$, $\tau_{2}=\left(0.06,0.1\right)$, $\tau_{3}=\left(0.3,0.5\right)$, $\tau_{4}=\left(0.3,0.5\right)$, $\tau_{5}=\left(0.8,1\right)$, $\tau_{6}=\left(0.8,1\right)$, $\tau_{7}=\left(2,8\right)$, $\tau_{8}=\left(3,8\right)$, and $\tau_{9}=\left(3,8\right)$. For the virtual task, we use the parameter of $v_{i}=\left(1,8\right)$, that is, its execution time at the lowest frequency and the invocation period is 1 and 8 s, respectively.

### 5.2. Simulation Results

#### 5.2.1. Comparison to the Conventional Low-Power Mapping

**Figure 4:**
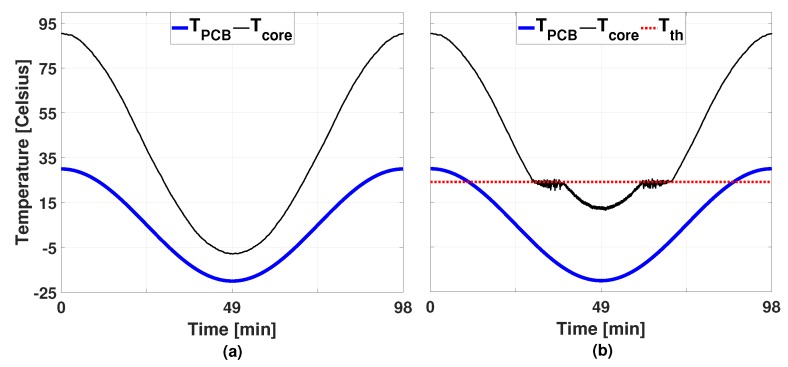
Temperature profiles of $T_{core}\left[0\right]$ caused (**a**) by Xian et al. [34] and (**b**) by the proposed technique without virtual task injections.

We first compare the proposed technique with the conventional low-power mapping approach. We choose Xian et al.’s work [34] as a comparable target which is based on the WF bin packing heuristic that balances the load to minimize the total energy consumption. The mapping decision is made in each hyper-period, that is 8 seconds and the EDF scheduling policy is adopted for the scheduling of the multiple workloads assigned to the same core. Figure 4a depicts the resultant temperature profile of Xian et al. [34], which causes a large TC with an amplitude of about 98 degrees. In this case, the average power consumption is 3.79 W.

We first apply the proposed technique without virtual task injections (lines 10–13 of Algorithm 3) and the resultant temperature trace is shown in Figure 4b. As can be seen in the figure, applying DVFS solely is not enough to prevent the temperature threshold violations from happening at the lower ambient temperatures. However, despite that, the normalized MTTF has been improved by 5.48 times as summarized in Table 1. Due to the artificial heat up procedure (Algorithm 3), the average power consumption has increased to 4.59 W.

#### 5.2.2. Different Power Budgets

**Figure 5:**
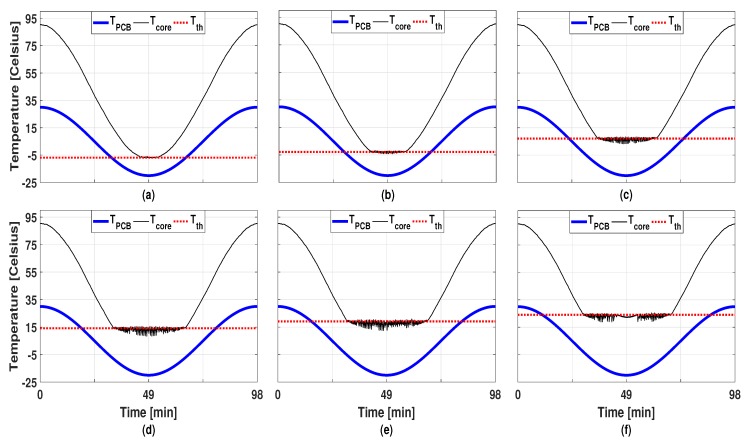
Temperature profiles asb of $T_{core}\left[0\right]$ with the proposed technique using virtual tasks and DVFS under different power constraint: (**a**) 4.02 W, (**b**) 4.08 W, (**c**) 4.34 W, (**d**) 4.59 W, (**e**) 4.81 W, and (**f**) 5.00 W.

In order to investigate the effects of the power budget in the proposed technique, we try six different power budgets (4.02 W, 4.08 W, 4.34 W, 4.59 W, 4.81 W, and 5.00 W), and, in this case, the virtual task injection is enabled. Figure 5 shows the temperature profiles caused by each case and Table 1 summarizes the threshold temperature, the average power consumption, and the normalized MTTF. Due to space limitation, only the temperature profiles for one core ($T_{core}\left[0\right]$) are displayed in Figure 5 and Table 1. It is clearly noticeable that a bigger power budget allows for a higher threshold temperature. Thanks to the higher threshold, the amplitude of the TC could be effectively reduced, bringing the gain of enlarged MTTF values of up to 8.03 times. In all cases, the average power consumption was kept below the imposed power budget as summarized in Table 1.

#### 5.2.3. Effect of Frequency Ranges

If the hardware supports a wider frequency range, it has more room for lifetime enhancement. In the setup we used in the previous experiments when the frequency scaling is possible up to 2.52 GHz, the normalized MTTF is further enhanced to 22.15 with the average power consumption of 5.56 W. In modern satellite systems, however, it is not common to have such high frequency. In the case of narrower ranges, the proposed technique could considerably maximize the MTTF. When the maximum frequency is set to 1.73 GHz, the normalized MTTF is 1.21 with the average power consumption of 3.87 W.

#### 5.2.4. Effect of Coffin-Manson Exponent

Lastly, we show how sensitive the proposed technique to the parameters of the Coffin-Manson equation. As mentioned in Section 3.3, the Coffin-Manson exponent *q* is material dependent and the effect of TC is significantly dependent on the *q* value. In order to quantify the effect of this value, we compare the two TCs presented in Figure 3, varying *q* from 7 to 2. The MTTF gains of the cycle with a smaller amplitude ($\left[T_{top},T_{bot,max}\right]$) to the other one ($\left[T_{top},T_{bot,min}\right]$) were 17.50, 11.42, 7.50, 4.91, 2.21, and 0.52, respectively for q=7, q=6, q=5, q=4, q=3, and q=2. When *q* is relatively big, the MTTF gain is more significant. Conversely, a very small *q* value, q=2 in this case, the reduced amplitude may result in a negative effect in MTTF. This is because that the adverse effects in EM, TDDB, and SM, due to the increased temperature, nullify the gain in TC. However, we could observe a considerable gain in MTTF even with a considerably small *q* value, that is, 2.21 X gain in MTTF with q=3. The typical choice of *q* is 6–9 for brittle fracture (Si and dielectrics), 3–5 for hard metal alloys/intermetallics (Al-Au), and 1–3 for ductile metal (solder) [18]. Therefore, we believe that the proposed technique is effective for the most materials used in the IC and package of the multi-core microprocessors.

## 6. Conclusions and Future Works

In this paper, we presented a lifetime enhancement technique in multi-core satellite embedded systems using virtual tasks and DVFS under power constraints. In LEO CubeSats, the temperature changes greatly and a large TC occurs in the electronic parts, which is a well-known lifetime reliability threat. Conventional reliability enhancement techniques focus on lowering the operating temperature whenever possible, but in LEO CubeSats, the TC can be large and the MTTF may deteriorate. The proposed technique focuses on reducing a large TC amplitude by intentionally consuming more power while considering other high temperature-dependent failure mechanisms. The proposed technique manages the system temperature in three steps. First, it minimizes the peak temperature at the highest PCB temperature to reduce TC amplitude. Second, it derives the minimum temperature (threshold), above which the system’s temperature should be maintained, considering the power budget. Lastly, at runtime, whenever the temperature goes below the threshold, it artificially heats up the processor by means of DVFS and virtual task injections. Experimental results show that the proposed technique improves the MTTF up to 8.03 times in the Nvidia’s Jetson TK1 board with a real workload of a small satellite system. In the extension of the proposed technique of no virtual tasks, different frequency range and different Coffin-Manson exponent, we show that our proposed technique is effective in improving the MTTF.

The proposed technique can be further extended as follows to be better utilized in the small satellite systems. Firstly, it has been reported that spatial thermal gradients in the satellite system would result in negative effects in reliability [38]. As the reliability model that we used in this work only focuses on the temporal gradient, it is necessary to investigate how the spatial gradients, for example, between cores, can be analyzed and mitigated in mapping and DVFS. Another future work that can be done is to co-optimize the reliability and power consumption at the same time. Whilst the power budget was given as input and the lifetime is just to be maximized in this work, the mission lifetime can be different from one mission to another in reality. Thus, how much power to be sacrificed can be effectively balanced with the target lifetime during the optimization, which also remains as a future work.

## Figures and Tables

**Table 1:** Comparisons of $T_{th}$, average power consumption, and normalized MTTF.

Figure	4(a)	4(b)	5(a)	5(b)	5(c)	5(d)	5(e)	5(f)
$T_{th}\left[0\right]$ (${}^{\circ }C$)	−	24.11	−6.89	−2.89	7.11	14.11	19.11	24.11
Average Power (W)	3.79	4.59	3.81	3.89	4.11	4.35	4.53	4.81
Normalized MTTF	1	5.48	1.02	1.32	2.24	3.65	4.89	8.03

## Abbreviations

The following abbreviations are used in this manuscript:

COTS	Commercial-off-the-shelf
CubeSats	Cube satellites
LEO	Low earth orbit
TC	Thermal cycling
BAT	Battery
EXT	External
PCB	Printed circuit board
MB	Motherboard
FPGAs	Field-programmable gate arrays
GEO	Geostationary orbit
ICs	Integrated circuits
EM	Electromigration
TDDB	Time-dependent dielectric breakdown
SM	Stress migration
MTTF	Mean time to failure
RAMP	Reliability-aware microprocessor
NBTI	Negative bias temperature instability
SOFR	Sum-of-failure-rates
DPM	Dynamic power management
DVS	Dynamic voltage scaling
DTM	Dynamic thermal management
ECUs	Electronic control units
DVFS	Dynamic voltage and frequency scaling
EDF	Earliest-deadline-first
PWL	Piece-wise linear
WF	Worst-fit
